# A Wearable System to Objectify Assessment of Motor Tasks for Supporting Parkinson’s Disease Diagnosis

**DOI:** 10.3390/s20092630

**Published:** 2020-05-05

**Authors:** Erika Rovini, Carlo Maremmani, Filippo Cavallo

**Affiliations:** 1 The BioRobotics Institute, Scuola Superiore Sant’Anna, viale Rinaldo Piaggio 34, Pontedera, 56025 Pisa, Italy; filippo.cavallo@unifi.it; 2 Department of Excellence in Robotics & AI, Scuola Superiore Sant’Anna, Piazza Martiri della Libertà, 33, 56127 Pisa, Italy; 3 O.U. Neurology, Ospedale delle Apuane, AUSL Toscana Nord Ovest, via Enrico Mattei, 21, 54100 Massa, Italy; carlo.maremmani@uslnordovest.toscana.it; 4 Department of Industrial Engineering, University of Florence, via Santa Marta 3, 50139 Florence, Italy

**Keywords:** decision support system, motion analysis, motor assessment, Parkinson’s disease diagnosis, signal processing, supervised learning, wearable inertial devices

## Abstract

Objective assessment of the motor evaluation test for Parkinson’s disease (PD) diagnosis is an open issue both for clinical and technical experts since it could improve current clinical practice with benefits both for patients and healthcare systems. In this work, a wearable system composed of four inertial devices (two SensHand and two SensFoot), and related processing algorithms for extracting parameters from limbs motion was tested on 40 healthy subjects and 40 PD patients. Seventy-eight and 96 kinematic parameters were measured from lower and upper limbs, respectively. Statistical and correlation analysis allowed to define four datasets that were used to train and test five supervised learning classifiers. Excellent discrimination between the two groups was obtained with all the classifiers (average accuracy ranging from 0.936 to 0.960) and all the datasets (average accuracy ranging from 0.953 to 0.966), over three conditions that included parameters derived from lower, upper or all limbs. The best performances (accuracy = 1.00) were obtained when classifying all the limbs with linear support vector machine (SVM) or gaussian SVM. Even if further studies should be done, the current results are strongly promising to improve this system as a support tool for clinicians in objectifying PD diagnosis and monitoring.

## 1. Introduction

Worldwide Parkinson’s disease (PD) is the second most common neurodegenerative disorder. It is caused by both genetic and environmental factors and no definitive therapy is currently available to cure it. PD symptoms become evident when dopamine in the forebrain reaches a critical deficiency, i.e., when the death of cells that produce dopamine transmitters exceeds 60% [1]. The global burden of PD in the society is well-documented [2]; it is estimated that currently over 6 million people are affected by this pathology, which has an increasing prevalence rate, as is expected to reach about 9 million people by 2030 [3]. The numerous impairments characterizing PD include both motor [1] and non-motor [4] symptoms that are very disabling for patients. Therefore, quality of life (QoL) of persons with PD is hugely affected by the pathology development [5], that, besides, leads to increased caregivers’ burden. Moreover, the excessive burden can direct caregivers to burnout with consequently premature institutionalization of the PD patient [6]. Also, the costs related to the pathology, measured as direct, indirect and intangible costs, have a strong economic impact on patients, caregivers and national health systems [7,8]. The slowdown of the disease progression rate could allow a significant saving of money, producing economic benefits both to citizen and healthcare systems. Since costs are directly correlated to the severity of the disease (i.e., low costs for mild patients, high costs for advanced patients) [7], the opportunity to detect PD in an early phase is crucial to provide optimal treatment and improve patients QoL [9], while misdiagnosis or late diagnosis can cause delays in applying the most appropriate treatment to the patients.

Although the Movement Disorders Society (MDS) recently updated the diagnostic criteria for PD [10,11], introducing also the non-motor symptoms as additional diagnostic features and dealing with the prodromal phase of the pathology, these revised criteria are still scarcely employed among clinicians [12].

Thus, currently, PD diagnosis is mainly based on clinical criteria and neurological examination of the patients (or suspected patients) administering them the MDS−Unified Parkinson’s Disease Rating Scale (MDS-UPDRS) [13]. In particular, Section III of the MDS-UPDRS is dedicated to investigating and assessing the motor capabilities of the patient according to the execution of standardized tasks. In practice, the neurologist asks the patient to carry out specific motor tasks that mainly involve upper and lower limbs and observes him/her. For each task, a score ranging from 0 (no motor signs) to 4 (severe motor sings) is assigned by the neurologist and the sum over all the tasks returns the evaluation of the patient. Well-known problems are related to this type of assessment; first of all, the evaluation strongly depends on the patient’s status at the moment of the examination [14] and furthermore, it is also biased by the subjectivity of the clinical expert that assesses the patient, thus the evaluation can be affected by high inter-rater variability [15].

Considering the lack of objective assessment of patients’ motion capabilities when evaluated during clinical examinations, many attempts were made in the last decade to introduce novel technologies in clinical practice to provide neurologists with reliable tools for objectively measuring motor impairments in PD patients. The importance and potential of wearable systems and machine learning algorithms to develop useful decision support systems for improving the assessment of motor symptoms related to PD have been investigated and proved in several studies [3,16,17]. Nonetheless, an effective application of technological systems in day-to-day practice for supporting PD diagnosis and monitoring has not been achieved yet. 

From the literature review, it is apparent that many studies have investigated and pursued the use of wearable sensor devices, together with machine learning techniques, for different applications, including indoor navigation [18], activity recognition [19] and objective assessment of PD motor symptoms [3,15,16]. Concerning PD evaluation, limited datasets and issues about acceptance, usability, reliability, and accuracy of the technological solutions represent the main limitations of most of those works. Moreover, most of the studies focused on a single symptom (e.g., tremor, bradykinesia), or analysed a single task, with a prevalence of gait analysis [15,16].

Finally, in the last three years, more attention has also been given to the analysis of the movement of the upper limbs. For example, Garza Rodriguez et al. [20] analysed in depth the pronation-supination movement of the hands, using a fuzzy inference system to process the information obtained from eight features for calculating a score between 0–4 to be compared to the UPDRS. The results showed good agreement with evaluations provided by neurologists. However, the system was only able to record data from the wrists, appearing inadequate for the fine motor assessment of fingers that typically are examined for PD evaluations. Differently, Bobic et al. [21] proposed a decision support system based on the analysis of the finger tapping exercise only. Five parameters were extracted from this task and correlation with the clinical assessment provided by the neurologist was investigated. The system achieved a good agreement with neurologists’ diagnosis, but the dataset used included small groups (i.e., less than 20 people per group). Moreover, the device is not presented, thus it is difficult to understand if it could be robust, reliable, and available for use in clinical practice. The same group [22] already published prior results about the objective measurement of kinematic parameters from finger-tapping exercise comparing measurements from the wearable sensor to those obtained from a gold standard optical system. Good results were obtained using two different algorithms (named AL-R and AL-C) reporting a root mean square error (RMSE) less than 4° (3.16° AL-R, 3.98° AL-C) and an interclass correlation coefficient (ICC) between 0.972 (AL-R) and 0.980 (AL-C). Nonetheless, also in this paper, it is not clear if the device could be available for real use. Finally, Van den Noort et al. [23] also proposed a system, called Power Glove, to quantify the hand motor symptoms, proposing a set of exercises and numerous parameters to measure. However, this was just a proof of concept that enrolled 4 PD patients only, thus statistical validity is not achievable. Furthermore, looking at the images, the system seems a prototype with low technological readiness level (TRL) [24], and further studies about its development are not available to the best of our knowledge.

Also, analysis of foot-tapping by using wearable inertial sensors is not receiving much attention in the last three years, which is unexpected since lower limbs tasks like toe-tapping and leg agility are included in the MDS-UPDRS III.

Only very recently the idea that using a fusion of data coming from both upper and lower limbs could improve the accuracy in PD motor states estimation is arising accomplished by encouraging preliminary results [25,26,27,28].

In this context, this work aims at proposing a wearable system composed of four inertial devices for upper and lower limbs analysis and custom-made algorithms able to extract a wide number of parameters to objectively characterize the motor performance of investigated people. A preliminary version of this work was published in [26], where a small number of patients was considered and minor investigations with machine learning techniques were implemented. Differently, this paper shows in detail how to process the raw inertial data acquired to calculate many features that can support the neurologist in a decision-making process for objective diagnosis and assessment of Parkinson’s disease. The idea is to overcome the traditional evaluation of single symptoms or impairments (i.e., bradykinesia, tremor, dyskinesias) in favour of an integrated assessment of the people motor capabilities. For this achievement, the use of techniques of artificial intelligence able to aggregate, interpret and classify the big amount of information acquired by the inertial devices is unavoidable. Appropriate machine learning algorithms can provide an overview of the examined subjects, however, allowing to the neurologist the possibility, if needed, to retrieve information about a single exercise or a single parameter. In this paper, the application of several machine learning approaches over eighty subjects wants to represent proof of how the numerous extracted features are important and reliable for identifying motor impairments caused by PD. Therefore, this work wants to focus on the processing algorithms of the data obtained from the wearable inertial sensors to characterize the motor performances of subjects undergoing the motor evaluation protocol. The paper points out the computation of many parameters from spatio-temporal and frequency domains that surely can improve the visual examination of the neurologist, providing additional information that cannot be caught during the traditional evaluation motor test. In this context, the system could improve the PD diagnosis thanks to the numerous, accurate, and useful information provided. Moreover, the opportunity to have this information while doing the clinical exam and record it allows creating a sort of database for each patient. Therefore, the clinician can have an objective assessment of reference for the next examinations. In this sense, the system could be used also for monitoring disease progression.

## 2. Materials and Methods

### 2.1. Participants

Eighty people were enrolled for this study: 40 healthy control subjects (HC) (34 males, six females, mean age ± standard deviation [SD] 65.6 ± 2.6 years old), and 40 PD patients affected by Parkinson’s Disease (PD) (25 males, 15 females, mean age ± SD 66.4 ± 9.1 years old). The PD patients were mild to mid, according to the clinical assessment based MDS-UPDRS III and Hoehn & Yahr (HY) scales (mean MDS-UPDRS III ± SD score: 15.1 ± 7.7; mean HY ± SD score: 1.9 ± 0.7; mean L-dopa equivalent dose ± SD: 463.7 ± 313.3 mg), with a prevalence of the disease almost equally distributed (13 unilateral right, 12 unilateral left, and 15 bilateral). The two cohorts were matched in age.

The design of the study agrees with the Declaration of Helsinki, and the experimental protocol was approved by the Ethical Committee of Massa and Carrara Local Health Institution (No. 311, 18/11/2010). Informed written consent was obtained from all individual participants included in the study. Subjects that manifested impairments or diseases, other than PD, that could affect motor performance during daily activities (e.g., orthopaedic injuries, neurological disorders) were excluded from the study. Further, all measurements on PD patients were acquired in a clinically defined ON-state.

### 2.2. Instruments

A system composed of four wearable devices based on inertial measurement units (IMUs) was used to collect and objectively analyse the motor performance of the subjects involved in the experimental test. The devices were developed and patented by the Assistive Robotics Lab of The BioRobotics Institute of Scuola Superiore Sant’Anna by Cavallo et al. [29].

**Figure 1:**
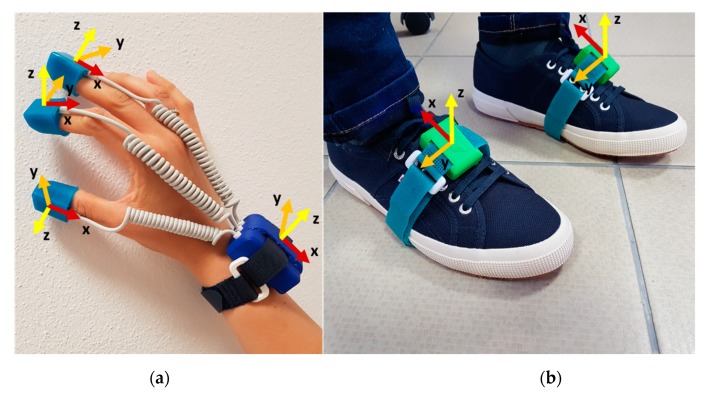
The wearable system: (**a**) SensHand for the right hand; (**b**) the two SensFoot. The axes of inertial sensors are reported for each unit of the system (*x*-axis in red, *y*-axis in orange, *z*-axis in yellow).

The system is low-cost, low-power, non-invasive, small, lightweight, wireless and easy to use. Both the devices are supplied by rechargeable LiPo batteries and they enable to collect data with 100 Hz sampling frequency. Two SensHand devices were used for the motion analysis of the upper limbs [30]. Each SensHand is composed of four modules, each of them equipped with an iNEMO-M1 board with dedicated STM32F103RE microcontrollers (ARM 32-bit Cortex™-M3 CPU, STMicroelectronics, Agrate Brianza, Monza and Brianza, Italy) and based on MEMS sensors that include 3-axis digital gyroscope L3G4200D (user-selectable angular rate full-scale of 250/500/2000 deg/s, finally set on 2000 deg/s, ST Microelectronics, Italy) and a ST Microelectronics 6-axis geomagnetic module LSM303DLHC (dynamically user-selectable full-scale acceleration range of 2/4/8/16 *g* and a magnetic field full-scale of 1.3/1.9/2.5/4.0/4.7/5.6/8.1 gauss, finally set on 8 *g* and 4.7 gauss, respectively). The module placed on the wrist acts as the coordinator of the system and it is equipped also with a Parani™-ESD210 (Sena Technologies, Inc., Irvine, CA, USA) Bluetooth serial device for wireless communication towards a control station. The other modules are placed on the distal phalanges of the thumb, index and middle fingers, included in silicon finger stalls printed with different sizes to be adapted for each subject. Modules coordination and data synchronisation are implemented through the controller area network (CAN-bus) standard (Figure 1a).

For the analysis of the lower limbs, instead, two SensFoot devices were adopted [31]. Each SensFoot consists of an IMU integrated into the iNEMO-M1 board based on MEMS sensors (L3G4200D 3-axis gyroscope set on 2000 deg/s, and LSM303DLHC 6-axis geomagnetic module set on 8 *g* and 4.7 gauss) and a STM32F103RE ARM-based 32-bit microcontroller (ST Microelectronics). The system is integrated with a SPBT2632C2A Bluetooth module (v.3.0, STMicroelectronics) which wirelessly transmits data acquired to a remote personal computer for offline analysis. The device is placed on the dorsum of the subject’s foot within a Velcro strap to ensure no movements occur between foot and sensor (Figure 1b).

Both the SensFoot devices and the coordinators of SensHand are included in a plastic cover realized using a 3D printing technique. Data are collected on a PC through a custom-made interface developed in Visual Studio, C# language.

### 2.3. Experimental Protocol

The experimental protocol to assess the motor performance of the subjects was derived from the tasks described in Section 3 of the MDS-UPDRS. Eleven exercises were proposed in this work, and they included tasks for upper limbs, lower limbs, and a task, i.e., the gait, during which the four limbs were simultaneously analysed. In addition to the standard tasks, two further tapping exercises were proposed for the analysis of the lower limb motor abilities.

Before starting the experimental session, each subject was asked to observe the correct execution of each exercise as explained and showed by the clinical staff who administered the test. A brief training to repeat all the movements was required for each subject to ensure that everyone had understood how to correctly perform the exercises. During the experimentation, the clinician indicated the exercises to be carried out, from time to time, before acquiring them, but no information was provided to the subjects while they were performed the tasks.

A specific initial fixed position was established for each exercise to allow 3 seconds of static acquisition, to have a baseline for each trial; then a beep from the PC indicated to start the exercise. All the exercises lasted 10 s, except for the gait and rotation that finished when the subjects had covered the required distance or angle, respectively. At the end of the exercises, further 3 s of static acquisition were recorded.

For all the upper and lower limb tasks, except for rotation, the subjects assumed a comfortable sitting posture (i.e., right angles at the hip, between trunk and thighs, and at the knees, between thighs and shins); differently, they stayed standing with the arms at their sides for the gait and rotation exercises. Subjects performed each exercise twice, to have two acquisitions from both the right and the left sides. For comparison between groups, the average values of the two acquisitions were considered. In the following sub-paragraphs, all the exercises are explained in detail and a video to clarify how the tasks are performed is available as Supplementary Materials.

#### 2.3.1. Upper Limb Tasks

- Thumb–Forefinger Tapping (THFF): the subject kept the hand fixed on the desk so that the plane between the thumb and forefinger joined was parallel to the table. In the starting position, the thumb and the forefinger were in contact, then the subject tapped the forefinger against the thumb as quickly and widely as possible (MDS-UPDRS 3.4—*Finger tapping*)
- Hand Opening/Closing (OPCL): the subject flexed the arm that was fixed on the table at the elbow, keeping the palm in front of him/herself. Starting from a fist position, the subject had to alternately open and close the hand, holding the forearm and the wrist fixed as quickly and widely as possible (MDS-UPDRS 3.5—*Hand movements*).
- Hand Pronation/Supination (PSUP): the subject put the arm outstretched in front of himself, with the wrist stable and the hand in prone position. The pronosupination movements had to be performed in parallel to the floor as quickly and widely as possible (MDS-UPDRS 3.6—*Pronation-supination movements of hands*).
- Hand Resting Tremor (HRST): the subject put the hand on the table in the prone position. This position has kept for the duration of the exercise, with the hand fully relaxed, without contrasting the potential tremor. Additionally, as a distracting task to get evidence on the potential tremor, they were asked to count backwards (MDS-UPDRS 3.17—*Hand rest tremor amplitude*).
- Hand Postural Tremor (POST): the subject put the arm outstretched in front of himself, with the wrist stable, the hand in the prone position and the fingers outstretched for all the time (MDS-UPDRS 3.15—*Postural tremor of hands*).

#### 2.3.2. Lower Limb Tasks

- Toe Tapping Heel Pin (TTHP): the subject tapped the toe on the floor always keeping the heel in contact with the ground, as quickly and widely as possible (MDS-UPDRS 3.7—*Toe Tapping*).
- Leg Agility (HEHE): the subject tapped the heel on the floor always keeping the forefoot raised from the ground, as quickly and widely as possible (MDS-UPDRS 3.8—*Leg agility*).
- Heel Tapping Toe Pin (HTTP): the subject tapped the heel on the floor always keeping the toe in contact with the ground, as quickly and widely as possible.
- Heel-Toe Tapping (HETO): the subject tapped alternatively the heel and the toe on the floor, as quickly and widely as possible.
- Rotation (ROTA): the subject turned once in the clockwise direction for 360°, then the rotation has repeated in the anticlockwise direction (part of MDS-UPDRS 3.10—*Gait*).

#### 2.3.3. Full Task

- Gait (GTAF + GTAH): the subject started the gait with the right foot, walking 15 m linearly until reached the finish line. The subject had to walk most naturally, at the preferred velocity. Both walking (GTAF) and arms swinging (GTAH) are simultaneously acquired and analysed (part of MDS-UPDRS 3.10—*Gait*).

Therefore, the protocol included movement tasks (e.g., ROTA, GTAF, GTAH), many of which are repetitive exercises (i.e., THFF, OPCL, PSUP, TTHP, HTTP, HETO, HEHE), and tremor tasks (i.e., HRST, POST). The full list of the abbreviations used in this paper is reported in Appendix A, Table A1.

### 2.4. Signal Processing

The motor data recorded with SensFoot and SensHand were stored on a PC during the acquisitions and offline processed by using Matlab^®^R2019b (The MathWorks, Inc., Natick, MA, USA). Accelerometers and gyroscopes provided triaxial accelerations and triaxial angular rates, respectively, that were processed to measure kinematic parameters.

#### 2.4.1. Pre-Processing

##### Movement Tasks

For all movement tasks (i.e., all exercises excluded HRST and POST), a fourth-order low-pass digital Butterworth filter was applied (the cut-off frequency is equal to 5 Hz) to acceleration and angular rate data to remove high-frequency noise and tremor frequency bands [32], typically identified between 3.5–7.5 Hz [33]. The choice for a 5-Hz cut-off frequency represents a trade-off between removing pathological tremor while preserving significant information from the acquired signals. Indeed, a cut-off frequency of less than 5 Hz could delete useful information when the exercise is executed particularly fast. On the other hand, a cut-off frequency higher than 5 Hz could maintain tremor noise together with the useful signal when Parkinsonian tremor occurs. Differently, for gait, the cut-off frequency was fixed to 3 Hz, since the cadence during a free walking in this sample is typically around 1Hz.

##### Tremor Tasks

For HRST and POST, which aimed at investigating tremor, 15 Hz and 20 Hz as cut-off frequency were respectively chosen. A further fourth-order high-pass digital Butterworth filter was applied (cut-off frequency equals to 0.5 Hz) for removing the continuous component of the signal.

#### 2.4.2. Segmentation and Events Detection

For each movement exercise, the most informative signals were identified for processing. For example, for THFF, the angular velocity of the index finger that was orthogonal to the plane where the tapping movement occurred was selected (i.e., ω_y_). Differently, for PSUP the angular velocity of the wrist that was aligned with the forearm was selected (i.e., ω_x_). Concerning the foot-tapping exercises (i.e., TTHP, HTTP, HETO) the angular velocity orthogonal to the movement was chosen (i.e., ω_y_). Also, the GTAF was analysed in the sagittal plane (i.e., considering *a*_x_, *a*_z_, ω_y_), which is the direction of the motion, while ROTA was referred to the vertical axis *z*.

Then, the selected signals were segmented, identifying characteristic times that delimit typical patterns in the signal according to the type of task. For example, for repetitive exercises for which angular velocities were selected as representative of the movements (i.e., THFF, OPCL, PSUP, TTHP, HTTP, HETO), three characteristic times were defined (i.e., when the action starts, when the movement reaches the maximum amplitude, and when the action ends), thus the movements were divided into two phases: the opening and the closing phase. Differently, for gait analysis the typical gait cycle phases were identified according to [34], identifying four characteristic times (i.e., when the foot starts to move, when the toe-off from the ground, when the heel strikes the ground and when the foot is completely flat) and the related static and swing phases. Different thresholds were set among the different exercises to segment the inertial signals. Indeed, fine and coarse tasks are characterized by different dynamics, with different execution velocity and different movement amplitude. Therefore, all tasks cannot be analyzed in the same way and different threshold values have appeared necessary.

##### Movement Tasks

- THFF

T_start_: it is the start of the tapping when the forefinger tip moves away from the thumb finger. It is assumed that this transition occurs when ω_y_(t) ≥ TH_TF_, where TH_TF_ = 15°/s is a threshold of the angular velocity to identify the beginning of the movement.

T_TF_: the maximum amplitude of the movement has been reached and forefinger and thumb are at the maximum distance. This transition occurs when ω_y_(t) < 0°/s and the angular velocity changes its direction (i.e., from clockwise to anticlockwise).

T_end_: the movement is completed, and the forefinger tip contacts the thumb again. This transition occurs when ω_y_(t) ≥ TH_TFv_, where TH_TFv_ = −3°/s is an empiric threshold of the angular velocity to identify the index is stable again.

- OPCL

T_start_: it is the start of the movement when the fingers move away from the palm. It is assumed that this transition occurs when ω_y_(t) ≥ TH_OC_, where TH_OC_ = 30°/s is a threshold of the angular velocity to identify the beginning of the movement.

T_OC_: the maximum amplitude of the movement has been reached and the hand is completely open. This transition occurs when ω_y_(t) < 0°/s, the angular velocity changes its direction (i.e., from clockwise to anticlockwise direction) and the hand starts to be closed.

T_end_: the movement is completed, and the hand is fully closed in a fist. This transition occurs when ω_y_(t) ≥ TH_OCv_, where TH_OCv_ = −3°/s is an empiric threshold of the angular velocity to identify the fingers are stable.

- PSUP

T_start_: it is the start of the movement when the forearm starts to rotate. It is assumed that this transition occurs when ω_x_(t) ≥ TH_PS_ for the right side (or ω_x_(t) ≤ −TH_PS_ for the left hand), where TH_PS_ = 50°/s is a threshold of the angular velocity coaxial to the forearm to identify the beginning of the movement.

T_PS_: the maximum amplitude of the movement has been reached and supination is completed. This transition occurs when ω_x_(t) < 0°/s for the right side (or ω_x_(t) > 0°/s, for the left hand), the angular velocity changes its direction (i.e., from clockwise to anticlockwise direction or vice versa) and forearm starts the pronation movement.

T_end_: the movement is completed, with forearm and hand prone. This transition occurs when ω_x_(t) ≥ TH_PSv_ for the right side (or ω_x_(t) ≤ −TH_PSv_ for the left hand), where TH_PSv_ = −5°/s is an empiric threshold of the angular velocity to identify the forearm is stable.

- GTAH

T_start_: it is the start of the movement when the arm starts to swing. It is assumed that this transition occurs when ω_z_(t) ≥ TH_GT_, or ω_z_(t) ≤ −TH_GT,_ according to the case in which the first movement of the arm is a forward or backward motion, respectively, for the right arm, or the contrary for the left arm. TH_GT_ = 10°/s is a threshold of the angular velocity orthogonal to the arm that identifies the beginning of the movement.

T_front_: the arm has reached the maximum distance from the frontal plane of the body in the forward direction. This transition occurs when ω_z_(t) ≤ 2°/s for the right arm (or ω_z_(t) ≥ −2°/s for the left side), and the angular velocity changes its direction (i.e., from clockwise to anticlockwise or vice versa).

T_back_: the arm has reached the maximum distance from the frontal plane of the body in a backward direction. This transition occurs when ω_z_(t) ≥ −2°/s for the right arm (or ω_z_(t) ≤ 2°/s for the left side), and the angular velocity changes its direction (i.e., from anticlockwise to clockwise or vice versa).

- GTAF

T_start_: it is the start of walking when the heel moves away from the ground. It is assumed that this transition occurs when ω_y_(t) < −TH_HO_, where TH_HO_ = 50°/s is a threshold of the angular velocity to identify the raising of the heel from the ground.

T_TO_: only the toe gets in touch with the ground. This transition occurs when ω_y_(t) > 0°/s, the toe is going to be off from the ground (i.e., toe-off time), and the angular velocity changes its direction (i.e., from anticlockwise to clockwise).

T_HS_: the heel gets in touch again with the ground (i.e., heel-strike time). This transition occurs when ω_y_(t) < 0°/s and the angular velocity changes its direction (i.e., from clockwise to anticlockwise).

T_end_: the movement is completed, and the foot is totally lying on the floor. This transition occurs when ω_y_(t) > −TH_FF_, where TH_FF_ = 3°/s is an empiric threshold of the angular velocity to identify the foot flat phase, i.e., when the foot is stable on the ground.

- ROTA

T_start_: it is the start of the movement when the foot was raised from the ground and the rotation starts. It is assumed that this transition occurs when ω_z_(t) ≤ −TH_RO_ when the rotation is in the clockwise direction (i.e., rotating on the right foot), or for ω_z_(t) ≥ TH_RO_ when the rotation is in the anticlockwise direction (i.e., rotating on the left foot). TH_RO_ = 50°/s is a threshold of the angular velocity approximately coaxial to the vertical axis of the body.

T_end_: the movement is completed, and the foot is totally lying on the floor. This transition occurs when ω_z_(t) ≥ −TH_ROv_ for the clockwise rotation (or ω_z_(t) ≤ TH_ROv_ for the anticlockwise rotation), where TH_ROv_ = 5°/s is an empiric threshold of the angular velocity to identify the forearm is stable.

- TTHP

T_start_: it is the start of the tapping when the toe moves away from the ground. It is assumed that this transition occurs when ω_y_(t) > TH_TO_, where TH_TO_ = 10°/s is a threshold of the angular velocity to identify the raising of the toe.

T_HS_: only the heel gets in touch with the ground, and the toe reaches the highest position from the floor. This transition occurs when ω_y_(t) < 0°/s and the angular velocity changes its direction (i.e., from clockwise to anticlockwise).

T_end_: the movement is completed, and the foot is totally lying on the floor. This transition occurs when ω_y_(t) > −TH_FF_, where TH_FF_ = 3°/s is an empiric threshold of the angular velocity to identify the foot flat phase.

- HTTP

T_start_: it is the start of the tapping when the heel moves away from the ground. It is assumed that this transition occurs when ω_y_(t) < −TH_HO_, where TH_HO_ = 10°/s is a threshold of the angular velocity to identify the raising of the heel.

T_TO_: only the toe gets in touch with the ground, and the heel reaches the highest position from the floor. This transition occurs when ω_y_(t) > 0°/s and the angular velocity changes its direction (i.e., from anticlockwise to clockwise).

T_end_: the movement is completed, and the foot is totally lying on the floor. This transition occurs when ω_y_(t) < TH_FF_, where TH_FF_ = 3°/s is an empiric threshold of the angular velocity to identify the foot flat phase.

- HETO

T_start_: it is the start of the tapping. Since the first tap can be done with the toe or with the heel (as the user prefers), it is assumed that this transition can occur when ω_y_(t) > TH_TO_ (i.e., toe-off case), or ω_y_(t) < −TH_HO_ (i.e., heel-off case), where TH_TO_ = TH_HO_ = 20°/s is a threshold for the angular velocity to identify the beginning of the movement.

T_HS_: only the heel gets in touch with the ground, and the toe reaches the highest position from the floor. This transition occurs when ω_y_(t) < 0°/s and the angular velocity changes its direction (i.e., from clockwise to anticlockwise).

T_TO_: only the toe gets in touch with the ground, and the heel reaches the highest position from the floor. This transition occurs when ω_y_(t) > 0°/s and the angular velocity changes its direction again (i.e., from anticlockwise to clockwise).

- HEHE

T_start_: it is the start of the tapping when the toe moves away from the ground. It is assumed that this transition occurs when ω_y_(t) > TH_TO_, where TH_TO_ = 10°/s is a threshold of the angular velocity to identify the raising of the toe.

T_HE_: only the heel gets in touch with the ground.

T_end_: the movement is completed, and the foot is totally lying on the floor. This transition occurs when ω_y_(t) > −TH_FF_, where TH_FF_ = 3°/s is an empiric threshold of the angular velocity to identify the foot flat phase.

##### Tremor Tasks

Since HRST and POST tasks consisted of a static acquisition, characteristic times were not searched. Only T_start_, 3 seconds after the beginning of the acquisition, and T_end_ after 10 s from the start, were identified.

#### 2.4.3. Feature Extraction

##### Movement Tasks

**Figure 2:**
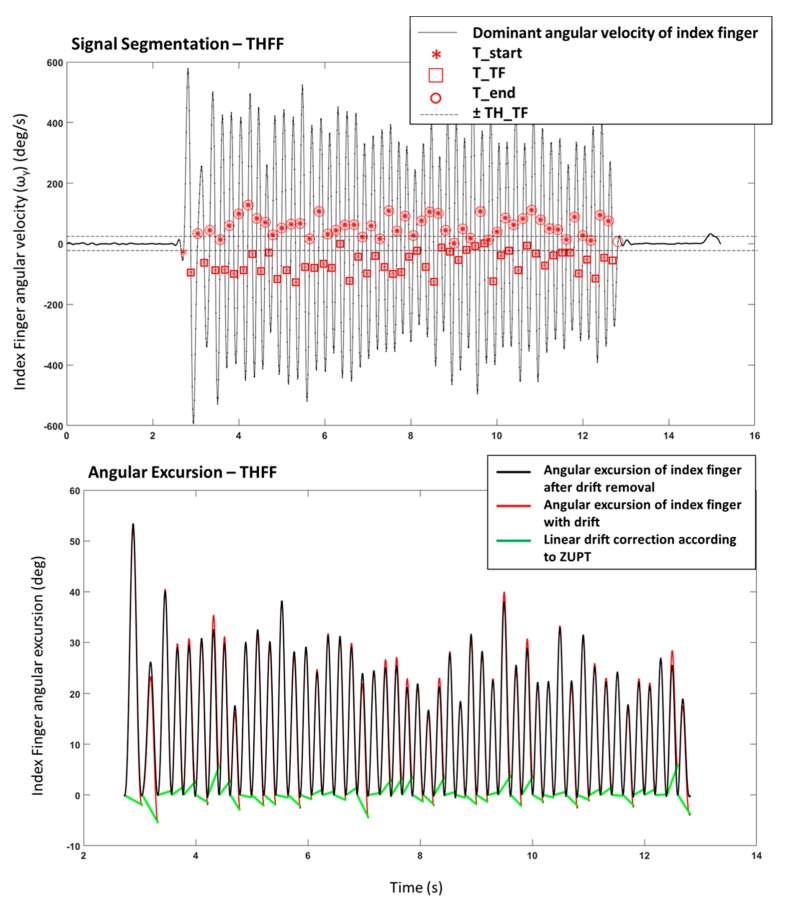
Example of a signal from THFF exercise. Upper panel: Signal Segmentation of the dominant angular velocity with identification of the characteristic times (T_start_, T_TF_, T_end_). Lower Panel: Angular Excursion obtained from the integration of the angular velocity with and without the linear drift correction.

Angular rates, selected and segmented during the previous step, were integrated to calculate the amplitude of the movements using the trapezoidal rule, with sub-intervals of integration equal to 100 ms, which is the inverse of the sensor-sampling rate. Linear drift correction was applied step by step to avoid cumulative effects, according to the theory of the zero velocity update (ZUPT) [35] (Figure 2). Thus, the correction at each step (e.g., each finger tapping, each step while walking) allow restraining the accumulation of error.

Based on the characteristic times, the following features were computed for TTHP, HTTP, THFF, OPCL, PSUP, GTAH:

- Number of movements:
  *Taps* = number of (*T_end_*), (1)
- Mean frequency:
  (2) $$Freq={\left(\frac{\sum_{i-1}^{Taps-1}\left(T_{end}\left(i+1\right)-T_{end}\left(i\right)\right)}{Taps-1}\right)}^{-1}$$
- Mean of the maximum movement amplitude (i.e., the maximum angle reached by the toe in the TTHP, maximum angle reached by the heel during HTTP, the maximum angular distance between finger and thumb in THFF, the maximum opening of the hand in OPCL, maximum excursion in supination movement in PSUP, the maximum swing of the arm in GTAH) over all the task:
  (3) $$Exc=\frac{\sum_{i=2}^{Taps-1}Exc(i)}{\left(Taps-2\right)},$$
  where:
  (4) $$\left\{ \begin{array}{c} Exc(i)=\max[\int_{T_{start}}^{j}\omega_{y}\left(t\right)dt-\tilde{\theta }\left(j\right)],\;\mathrm{for}\;\mathrm{TTHP},\;\mathrm{HTTP},\;\mathrm{THFF},\;\mathrm{OPCL}\\ Exc(i)=\max[\int_{T_{start}}^{j}\omega_{x}\left(t\right)dt-\tilde{\theta }\left(j\right)],\;\mathrm{for}\;\mathrm{PSUP}\\ Exc\left(i\right)=\max[\int_{T_{start}}^{j}\omega_{z}\left(t\right)dt-\tilde{\theta }\left(j\right)],\;\mathrm{for}\;\mathrm{GTAH} \end{array}\right.,$$
  with:
  (5) $$\tilde{\theta }\left(j\right)=[\frac{j-T_{start}}{T_{end}-T_{start}}]\cdot \theta \left(T_{end}\right).$$
- Integral of the magnitude of the total acceleration vector (IAV), which is related to the estimated energy expenditure [36]:
  (6) $$IAV=\int_{T_{start}}^{T_{end}}\sqrt{a_{x}^{2}+a_{y}^{2}+a_{z}^{2}}\;dt.$$
- Variability in frequency overall movements:
  (7) $$CVfreq=100\%\cdot \frac{\max[f_{j}]-\min[f_{j}]}{\max[f_{j}]},\;\mathrm{where}\;j=1,\ldots ,\mathrm{Taps}$$
  with:
  (8) $$f_{j}=\frac{1}{T_{end}(j+1)-T_{end}(j)}.$$
- Variability in amplitude overall movements:
  (9) $$\mathrm{CVexc}=100\%\cdot \frac{\max[{\mathrm{Exc}}_{j}]-\min[{\mathrm{Exc}}_{j}]}{\max[{\mathrm{Exc}}_{j}]},\;\mathrm{where}\;j=1,\ldots ,\mathrm{Taps}$$
- Mean of opening (or supination, or frontward) velocity overall movements (for TTHF, OPCL, PSUP, GTAH only):
  (10) $$\left\{ \begin{array}{c} \omega o=\frac{\sum_{i=2}^{\mathrm{Taps}-1}\omega o\left(i\right)}{\left(\mathrm{Taps}-2\right)},\;\mathrm{where}\;\omega o=\mathrm{mean}\left[\omega_{y}\left(j\right)\right],\;\mathrm{with}\;j=T_{\mathrm{Start}},\ldots ,\;T_{\mathrm{TF}}\left(\mathrm{or}\;T_{\mathrm{OC}}\right)\;\mathrm{for}\;\mathrm{THFF},\;\mathrm{OPCL}\\ \omega s=\frac{\sum_{i=2}^{\mathrm{Taps}-1}\omega s\left(i\right)}{\left(\mathrm{Taps}-2\right)},\;\mathrm{where}\;\omega s=\mathrm{mean}\left[\omega_{x}\left(j\right)\right],\;\mathrm{with}\;j=T_{\mathrm{Start}},\ldots ,\;T_{\mathrm{PS}}\;\;\mathrm{for}\;\mathrm{PSUP}\\ \omega f=\frac{\sum_{i=2}^{\mathrm{Taps}-1}\omega f\left(i\right)}{\left(\mathrm{Taps}-2\right)},\;\mathrm{where}\;\omega f=\mathrm{mean}\left[\omega_{z}\left(j\right)\right],\;\mathrm{with}\;j=T_{\mathrm{Start}},\ldots ,\;T_{\mathrm{front}}\;\;\mathrm{for}\;\mathrm{GTAH} \end{array}\right..$$
- Mean of closing (or pronation, or backward) velocity over all movements (for TTHF, OPCL, PSUP, GTAH only):
  (11) $$\left\{ \begin{array}{c} \omega c=\frac{\sum_{i=2}^{\mathrm{Taps}-1}\omega c\left(i\right)}{\left(\mathrm{Taps}-2\right)},\;\mathrm{where}\;\omega c=\mathrm{mean}\left[\omega_{y}\left(j\right)\right],\;\mathrm{with}\;j=\left(T_{\mathrm{TF}/\mathrm{OC}}+1\right),\ldots ,\;T_{\mathrm{end}}\;\mathrm{for}\;\mathrm{THFF},\;\mathrm{OPCL}\\ \omega p=\frac{\sum_{i=2}^{\mathrm{Taps}-1}\omega p\left(i\right)}{\left(\mathrm{Taps}-2\right)},\;\mathrm{where}\;\omega p=\mathrm{mean}\left[\omega_{x}\left(j\right)\right],\;\mathrm{with}\;j=\left(T_{\mathrm{PS}}+1\right),\ldots ,\;T_{\mathrm{end}}\;\;\mathrm{for}\;\mathrm{PSUP}\\ \omega b=\frac{\sum_{i=2}^{\mathrm{Taps}-1}\omega b\left(i\right)}{\left(\mathrm{Taps}-2\right)},\;\mathrm{where}\;\omega b=\mathrm{mean}\left[\omega_{z}\left(j\right)\right],\;\mathrm{with}\;j=\left(T_{\mathrm{front}}+1\right),\ldots ,\;T_{\mathrm{end}}\;\;\mathrm{for}\;\mathrm{GTAH} \end{array}\right..$$

The first and the last movements are excluded from the computation of the average amplitude and velocity of the movements. This choice is based on an extensive analysis of the used signal database, where it appears clearly that the first movement is often different from the others, often showing higher amplitude and velocity. On the other hand, the last movement is excluded as well because sometimes it appears incomplete, or highly reduced in amplitude when the subject receives the input of stopping the task and suddenly close the movement.

For the HETO task, some variations were considered for the computation of the parameters, as follows:

- Number of tapping movements:
  *Taps* = min[number(*T_HS_*); number(*T_TO_*)], (12)
- Mean frequency of the toe-tapping:
  (13) $$FreqT={\left(\frac{\sum_{i=2}^{Taps-1}\left(T_{TO}\left(i+1\right)-T_{TO}\left(i\right)\right)}{Taps-2}\right)}^{-1}$$
- Mean frequency of the heel tapping:
  (14) $$FreqH={\left(\frac{\sum_{i=2}^{Taps-1}\left(T_{HS}\left(i+1\right)-T_{HS}\left(i\right)\right)}{Taps-2}\right)}^{-1}$$
- Mean frequency of the heel-toe tapping:
  (15) $$FreqHT=\{\begin{array}{c} {\left(\frac{\sum_{i=2}^{Taps-1}\left(T_{HS}\left(i+1\right)-T_{TO}\left(i\right)\right)}{Taps-2}\right)}^{-1}\;\mathrm{if}\;T_{HS}\left(1\right)>T_{TO}\left(1\right)\\ \begin{array}{c} {\left(\frac{\sum_{i=2}^{Taps-1}\left(T_{TO}\left(i+1\right)-T_{HS}\left(i\right)\right)}{Taps-2}\right)}^{-1}\;\mathrm{if}\;T_{HS}\left(1\right)<T_{TO}\left(1\right) \end{array} \end{array}$$
- Mean of the maximum toe movement amplitude (i.e., the maximum angle from the ground reached by the toe) over all the taps:
  (16) $$ExcT=\frac{\sum_{i=2}^{Taps-1}ExcT\left(i\right)}{\left(Taps-2\right)},$$
  where:
  (17) $$ExcT\left(i\right)=\max\left[\int_{T_{TO}}^{T_{HS}}\omega_{y}\left(t\right)dt\right],$$
- Mean of the maximum heel movement amplitude (i.e., the maximum angle from the ground reached by the heel) over all the taps:
  (18) $$ExcH=\frac{\sum_{i=2}^{Taps-1}ExcH\left(i\right)}{\left(Taps-2\right)},$$
  where:
  (19) $$ExcH\left(i\right)=\max\left[\int_{T_{HS}}^{T_{TO}}\omega_{y}\left(t\right)dt\right]$$
- Variability in frequency overall movements as in Equation (7), where *f* is *FreqHT.*
- Variability in toe amplitude overall movements as in Equation (9), where *Exc* is *ExcT.*
- Variability in heel amplitude overall movements as in Equation (9), where *Exc* is *ExcH.*
- The IAV parameter was computed in the same manner of Equation (6).

For GTAF the following features were computed:

- Gait Time to cover 15 meters:
  *GT_Time* = *T_end_*(end) − *T_start_*(1). (20)
- Number of strides during 15 meters walking:
  *GT_Strd* = number(*T_end_*). (21)
- Mean Gait Frequency:
  *GT_Freq* = *GT_Strd/GT_Time* (22)
- Mean Stride Time:
  (23) $$GT\_StrdT=\frac{1}{GT\_Strd-1}\cdot \overset{GT\_Strd-1}{\underset{i=1}{\sum }}T_{HS}\left(i+1\right)-T_{HS}\left(i\right)$$
- Mean Swing Time:
  (24) $$GT\_SWT=\frac{1}{GT\_Strd}\cdot \overset{GT\_Strd}{\underset{i=1}{\sum }}T_{HS}\left(i\right)-T_{TO}\left(i\right)$$
- Mean Stance Time:
  (25) $$GT\_STT=\frac{1}{GT\_Strd}\cdot \overset{GT\_Strd}{\underset{i=1}{\sum }}GT\_StrdT\left(i\right)-GT\_SWT\left(i\right)$$
- Mean Relative Stance:
  (26) $$GT\_RS=\frac{100\%}{GT\_Strd}\cdot \overset{GT\_Strd}{\underset{i=1}{\sum }}\frac{GT\_STT\left(i\right)}{GT\_StrdT\left(i\right)}$$
- Mean of the maximum dorsiflexion angular excursion of the foot over all the strides:
  (27) $$GT\_Ang=\frac{1}{GT\_Strd-2}\cdot \overset{GT\_Strd-1}{\underset{i=2}{\sum }}\max\left(\theta \left(i\right)\right)-\min\left(\theta \left(i\right)\right)$$
  where:
  (28) $$\theta \left(i\right)=\int_{T_{start}}^{T_{end}}\omega_{y}\left(t\right)dt+\theta_{init},$$
  with:
  (29) $$\{\begin{array}{c} \theta_{init}\left(i=1\right)=\frac{1}{M}\cdot \overset{T_{start}}{\underset{k=T_{start}-M}{\sum }}\tan^{-1}\left(a_{x},a_{z}\right),\;\mathrm{where}\;M=10\\ \theta_{init}\left(i>1\right)=\frac{1}{N}\cdot \overset{T_{start}(i+1)}{\underset{k=Tend(i)}{\sum }}\tan^{-1}\left(a_{x},a_{z}\right),\;\mathrm{where}\;N=T_{\mathrm{start}}(i+1)-T_{end}(i) \end{array}$$

For ROTA the following features were computed:

- Rotation Time to cover 360 degrees:
  *RO_Time* = *T_end_*(end) − *T_start_*(1). (30)
- Number of strides during 15 meters walking:
  *RO_Strd* = number(*T_end_*). (31)
- Mean Gait Frequency:
  *RO_Freq* = *RO_Strd/RO_Time* (32)
- Total Stance Time:
  (33) $$RO\_STT=\overset{RO\_Strd-1}{\underset{i=1}{\sum }}T\_Start\left(i+1\right)-T\_end\left(i\right)$$
- Total Relative Stance:
  (34) $$RO\_RS=\frac{100\%\cdot RO\_STT}{RO\_Time}$$

##### Tremor Tasks

For HRST, POST, and HEHE, the analysis was performed in the frequency domain only. The Matlab function FFT that implemented the Fast Fourier Transform was applied to accelerometer and gyroscope signals, to obtain the Discrete Fourier Transform (DFT):

(35) $$X\left(i\right)=\overset{N}{\underset{j=1}{\sum }}X\left(j\right)\omega_{N}^{\left(j-1)(k-1\right)}$$

Then, Power Spectral Density (PSD) was computed as:

(36) $$P_{xx}\left(\omega \right)=\frac{1}{2_{\pi }}\overset{\infty }{\underset{m=-\infty }{\sum }}R_{xx}\left(m\right)e^{-j\omega m}$$

This equation, which is expressed as normalized frequency, can be written as a function of the physical frequency through the relation: *ω = 2πf*/*fs*, where *fs* is the sampling frequency:

(37) $$P_{xx}\left(\omega \right)=\frac{1}{f_{s}}\overset{\infty }{\underset{m=-\infty }{\sum }}R_{xx}\left(m\right)e^{-\frac{j2\pi mf}{f_{s}}}$$

Then, the average power of the signal in a certain frequency band [ω1, ω2] with 0 ≤ ω1 ≤ ω2 ≤ π is calculated as:

(38) $${\bar{P}}_{\left[\omega 1,\omega 2\right]}=\frac{1}{f_{s}}\int_{\omega 1}^{\omega 2}P_{xx}\left(\omega \right)d\omega$$

Starting from these formulae, specific Matlab functions were applied both to accelerometer and gyroscope signals to measure:

- Average power in PSD: it is the mean value of the power of the signal calculated through the PSD.
- Fundamental frequency: it is the frequency corresponding to the peak of the power.
- Maximum peak in PSD: it is the maximum value in the PSD of the acceleration signal, and it represents the peak of the power (for HEHE only)
- Percentage power of the signal in frequency band [3.5–7.5] Hz, which is typically associated with Parkinsonian tremor.
- Percentage power of the signal in frequency band [8–12] Hz, which is typically associated with physiological tremor (for POST only).
- The estimated energy expenditure, as IAV parameter, was computed in the same manner of Equation (6).

The complete list of parameters measured for each exercise is reported in Table 1 for lower limbs and in Table 2 for upper limbs. Parameters measured from the lower limbs were used to identify the FEET condition, while the parameters derived from the upper limbs constituted the HANDS condition. Then, all parameters were considered in the FULL condition.

### 2.5. Data Analysis

The parameters reported in Table 1 and Table 2 were measured for all the subjects, both for left and right side, then the possibility to distinguish the different groups of people involved in this study according to their motor performance was investigated. Particularly, 40 HC subjects versus 40 PD patients were analysed using binary classification.

Normality of data distribution was verified for each parameter applying the Kolmogorov– Smirnov test. Parameters calculated for the right side and left side were assessed separately because of the unilaterality that characterises the onset of the pathology. Since data from all extracted features were nonparametrically distributed, the nonparametric statistic tests were used. Statistical significance of each parameter was investigated by using the Wilcoxon rank-sum test for comparing two groups (i.e., HC vs. PD). The significance level was set at 5% (i.e., *p*-value < 0.05) and parameters resulted as significant were included in the post significance dataset (PS dataset). Because of the great number of comparisons, the Bonferroni correction was applied, controlling the familywise error rate (FWER) to avoid including features that can be significant by chance. Features from PS that remained significant after the Bonferroni correction were included in the PS_B dataset. However, both the solutions (i.e., including all the significant features after the Mann-Whitney test, and selecting only the significant features after the Bonferroni correction) were considered for the following steps of analysis.

#### 2.5.1. Feature Selection Process

A correlation analysis based on the Spearman’s correlation coefficients was implemented to reduce the feature space because of the great number of parameters included in the analysis. Both sides for upper and lower limbs were evaluated separately to identify parameters highly correlated with others. The cut-off correlation coefficient was set up at rho = 0.85 (and related *p*-value < 0.05), according to the methodology applied also in previous related works [26,30,31], and only the most significant parameter was maintained when two or more parameters resulted strongly correlated. Parameters from the PS dataset that resulted uncorrelated after the Spearman’s test were included in the post correlation dataset (PC dataset). Analogously, uncorrelated features from the PS_B dataset were included in the PC_B dataset. The dimensionality reduction is generally applied to avoid overfitting during the classification process.

#### 2.5.2. Classification

The ability of the selected features to correctly distinguish between HC and PD was evaluated applying supervised learning classifiers. In particular, support vector machine (SVM) with different kernel functions, random forest (RF), and naïve Bayes (NB) were trained and tested to implement the classification.

##### Support Vector Machine

SVM is particularly suitable when the data must be classified into two classes, by finding the best decision surface, called hyperplane, that separates all data points of one class from those of the other class. The best hyperplane is characterised by the largest margin between the two groups, and the support vectors are the closest elements to it. The function that generates the hyperplane depends on the choice of the kernel in the SVM model [37]. Types of kernels that can be implemented are linear, quadratic, Gaussian and polynomial. Main advantages of using SVM are that this technique has high predictive accuracy while tending not to overfit data and it is relatively easy to interpret. When applying SVM, there is need of time to generate the training model; once the model has been trained, the training data can be discarded if limited memory is available and the test is very fast.

The good generalization performance and the ability to provide good results when analysing a large set of attributes suggested that this technique could be suitable for our application. The SVM developed in this work implemented three different kernels: linear (SVM_L), Gaussian (SVM_G) and a third-order polynomial (SVM_P) kernel.

##### Random Forest

RF is an ensemble learning algorithm based on multiple decision trees constituting the forest. Each tree provides a binary decision and the majority vote over all the trees identifies the assignment of a new observation vector to a class. Main advantages of RF are that it is robust to noise and outliers and it has good performance even with large datasets. RF is based on a random step in the process of creating the trees and selecting a splitting feature [38]. Generally, default parameterization leads to excellent performance, thus the parametrization is quite simple [39]. Disadvantages of RF are that generating the model can take a lot of memory and the classification tends to overfit.

In this work, the package for MatLab [40] based on the Breiman et al. algorithm [38] was used to implement the RF classifier.

##### Naïve Bayes

NB are probabilistic learning algorithms, based on Bayes’ Theorem. They calculate the probability of each category for a given sample and then output the category with the highest probability. NB arises on the strong assumption of conditional independence of the features (i.e., independence of all attributes according to the value of the class variable) [41], thus NB performance can decrease when a high correlation between two or more features is found. Main advantages of NB are the ability to work well also with small datasets for training the model, and the quick training phase. Moreover, being simple, it does not tend to overfit data. On the other hand, when the dataset grows up in size and variance, the performance of NB decreases.

The parameters used to optimize the classifiers were automatically obtained from dedicated Matlab functions. For SVM the function fitcecoc was used activating the option about the ‘*HyperparameterOptimizationOptions*’. Analogously, for NB the function fitcnb was used activating the option about the ‘*HyperparameterOptimizationOptions*’. Differently, for RF the number of trees was varied using a base two exponential rate with the exponent from 1 to 12 [42] and the optimal number of trees was chosen according to a trade-off between the area under the curve (AUC) calculated from the receiver operating characteristic (ROC) and the corresponding processing time.

Multiple comparisons were evaluated in this work. In particular, 5 different classifications were implemented, and relative confusion matrices were calculated considering: (i) all the significant features (i.e., after the Mann-Whitney test, PS dataset); (ii) the corrected (i.e., after the Bonferroni correction) significant features (PS_B dataset); (iii) the significant uncorrelated (i.e., after the Spearman correlation) features (PC dataset); (iv) the corrected significant uncorrelated features (PC_B dataset). Furthermore, the motor performances of the subjects were assessed under three conditions, considering: (i) the parameters related to the lower limbs only (FEET); (ii) the parameters related to the upper limbs only (HANDS); (iii) all the parameters (FULL).

To minimize overfitting, the cross-validation model was implemented, evaluating the classifiers when predicting unseen datasets that were not used for training the system. 10-folds cross-validation was chosen, so that, each classifier was tested ten times and the average values were provided for evaluation metrics. Practically, the dataset of 80 subjects was randomly divided into 10 folds of eight subjects each. At each time, nine folds were used for training the model, and an unseen fold was used for testing it. Repeating the process 10 times, each fold was used for testing the system when not included for training. Confusion matrices reporting the results of classification were saved.

Finally, standard evaluation metrics were used to calculate the performance of the classifiers. True Positive (TP), False Positive (FP), True Negative (TP), and False Negative (FN) values were computed from the obtained confusion matrices. Then sensitivity or recall (i.e., percentage of cases that are correctly identified as true), specificity (i.e., percentage of cases that are correctly identified as false), precision (i.e., percentage of cases correctly identified as true concerning all predicted as true), accuracy (i.e., percentage of cases that are correctly identified over all subjects), and F-measure (i.e., a weighted average of the specificity and sensitivity) were obtained.

## 3. Results

In this section, results obtained from the multiple comparisons using the five supervised classifiers on four datasets (i.e., PS, PS_B, PC, and PC_B) evaluated over three conditions (FEET, HANDS and FULL) were reported in detail.

### 3.1. Feature Selection

Thirty-nine parameters were measured from the lower limbs for each side (i.e., a total of 78 parameters for the FEET condition), while 48 parameters were obtained from each side of the upper limbs (i.e., 96 parameters in total for the HANDS condition). Therefore, the FULL condition was composed of 87 features per side, which are overall 174. Since the parameters resulted in a non-parametric distribution, they were reported in Table 3 and Table 4 as median values and interquartile ranges (IQR) both for HC and PD. Moreover, that tables highlighted also what parameters were selected in each of the 4 defined datasets: PS, PS_B, PC, PC_B.

In PS dataset, the parameters selected per side were: 30 for FEET, 36 for HANDS, and 66 for FULL. Erasing the highly correlated parameters according to Spearman’s coefficients, the parameters resulted per side in PC dataset were: 18 for FEET, 28 for HANDS, 46 for FULL.

When applying the Bonferroni correction to reduce the FWER, the number of parameters included in the datasets decreased because the threshold on the *p*-value strongly decreased as well. However, a sizeable number of features per side remained: 25 for FEET, 23 for HANDS, and 48 for FULL in the PS_B dataset; 15 for FEET, 16 for HANDS, and 31 for FULL in the PC_B dataset.

It is worth noting that all the exercises contribute with at least one parameter to compose each dataset.

### 3.2. Classification Results

The four datasets (i.e., PS, PS_B, PC, PC_B) were used as input under the three conditions (i.e., FEET, HANDS, FULL) to train and test 5 different supervised learning methods to classify the subjects involved in this study according to their motor performance. The complete results derived from the evaluation metrics formulas are reported in Table 5 and Table 6.

**Figure 3:**
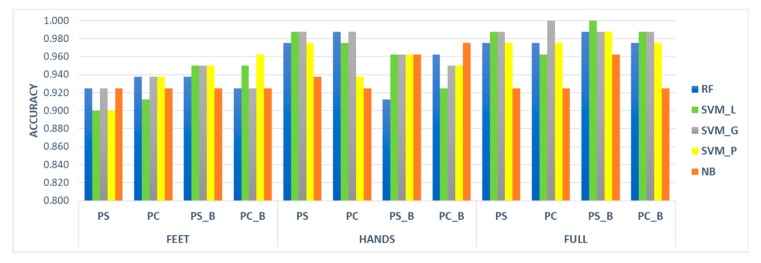
Accuracy index assessed by the five classifiers when applied to the four datasets over the FEET, HANDS, and FULL conditions.

All the classifiers showed excellent ability in distinguishing the two groups according to the motor performance analysis. Generally, results obtained under the FULL condition are better than those achieved when considering FEET and HANDS separately. In particular, FEET obtained the lowest results. Considering the accuracy index (see Figure 3), the worst case was represented by SVM_L and SVM_P using the PS dataset under the FEET condition. Nonetheless they both obtained accuracy equals to 0.900. The best cases, indeed, were achieved under the FULL condition by SVM_L tested on the PS_B dataset, and by SVM_G applied to the PC dataset with accuracies equal to 1.00.

Overall, the SVM_G reached the best performances in classifying HC and PD; however, minimum differences were found, on average, applying all the classifiers to all the datasets. Indeed, the average accuracy ranged from 0.936 for the NB to 0.966 for SVM_G. Also, analysing the four datasets, the average accuracy measured using all the classifiers was included between 0.953 for PS, PC, and PC_B and 0.960 for PS_B.

Such small differences in the accuracy index could be due to the specific dataset used in this work, even if 80 people can represent a sizeable sample for identifying significant parameters able to identify motor impairments caused by Parkinson’s disease.

## 4. Discussion

In this work, a wearable inertial sensor system, composed of the SensFoot and SensHand devices for motion analysis of lower and upper limbs is used aiming at quantifying and objectifying the traditional clinical evaluation of PD patients motor capabilities. The system, equipped with dedicated processing algorithms, was developed to provide a reliable and useful tool for the neurologist when assessing patients, or suspected patients, for PD, as well as for monitoring the motion performance of the patients throughout the development of the pathology. Many kinematic parameters were extracted from the inertial data acquired on 40 healthy subjects and 40 PD patients while performing a motor evaluation test. Statistical significance of the measured parameters was calculated with and without the Bonferroni correction; then dimensionality reduction of the dataset was implemented according to the results of the Spearman’s correlation. Finally, multiple comparisons among different supervised learning classifiers (i.e., RF, SVM_L, SVM_G, SVM_P, NB) were trained and tested on the four identified datasets (i.e., PS, PS_B, PC, PS_B) over three conditions (i.e., FEET, HANDS, FULL) to prove the consistency of the extracted selected features in identifying motor impairments caused by the Parkinsonian condition.

This work detailed the techniques of signal processing applied to the raw data acquired from the inertial sensors to extract a big number of kinematic parameters able to finely characterize the motor performance of a person when performing the specific tasks required for PD diagnosis evaluation and monitoring. An exhaustive protocol has been proposed in this study to provide a comprehensive characterization of the subjects motor skills, overcoming the idea, often found in the literature, of focusing on a single symptom, e.g., the tremor [33], or on a single exercise [20,21,22].

Since we aim at deploying this wearable system in clinical practice, the analysis of a reduced set of exercises could be insufficient both to correctly identify the pathology and to accurately characterize the patient condition. Indeed, PD has a typical asymmetric onset, with wide variability in clinical expression, as well as in the progression of somatic symptom, thus each subject should be investigated in each limb. This is the main reason that led us to consider separately the parameters derived from the right and the left side during the analysis. This approach allows to include surely the most affected side (together with the contralateral side), which is particularly important in this study where many patients are in the initial stages of the disease (average HY = 1.9), showing unilateral symptoms. Therefore, the total number of parameters measured for each subject involved in this study is 174.

Regarding the signal processing for the feature extraction, it is worth to note that the first and the last movements of each trial are excluded from the computation of the average amplitude of the movements (see Equations (3), (16), (18) and (27)). This choice is based on an extensive analysis of the used signal database, where it appears clearly that the first movement is often different from the others, showing often higher amplitude. On the other hand, the last movement is excluded as well because sometimes it appears incomplete, or highly reduced in amplitude when the subject receives the input of stopping the task and suddenly close the movement. These considerations were discussed together with the neurologist and clinical staff, those agreed with this choice. Indeed, even if the guidelines to assign the MDS-UPDRS score in tasks 3.4–3.8 talk about the analysis of ten repetitive movements, it is hard for the naked eye to catch and mentally analyse the different repetitions as distinct movements from the beginning. Therefore, generally, the score is assigned based on the overall evaluation of the task, because it is difficult to discriminate the single repetitions by visual inspection. For this reason, we also thought to acquire each task for 10 s, instead of 10 repetitions, aiming at obtaining more stable results.

Kinematic parameters obtained with the processing algorithms explained in detail in this work seem to be highly discriminant within our scope. Also considering the Bonferroni correction, which is notoriously very conservative and selective, a sizeable number of features resulted statistically significant. Similarly, the correlation analysis did not remove many parameters, because most of them are independent of each other. Finally, each dataset (i.e., PS, PS_B, PC, PC_B) is composed of one or more parameters from all the exercises included in the experimental protocol (Table 3 and Table 4). Therefore, the use of a large set of exercises and extracted parameters is not redundant but allow to acquire complementary information about the motor capabilities of a subject.

Interestingly, looking at the results obtained with the supervised learning techniques, there was not a classifier that outperformed the others (see Figure 3, Table 5 and Table 6). NB appeared as the slightly worse technique probably because of its characteristic of not working very well with big datasets; however, the results achieved by NB are widely satisfactory (the average accuracy over the four datasets equals 0.936). On the other hand, there was not a dataset among the four analysed that clearly resulted more significant than the others, with the average accuracy ranging from 0.953 for PS, PC, and PC_B to 0.960 for PS_B. This could be a proof of consistency for the extracted features; thus, they can represent a good pool of parameters (most of which are statistically significant) to correctly identify and quantify PD motor symptoms in a subject who was tested with SensHand and SensFoot. Furthermore, results obtained when investigating the FULL condition are generally better than those obtained when separately analysing FEET and HANDS, according to the results obtained on a reduced dataset in our previous work [26]. This is an additional demonstration that the use of the proposed comprehensive protocol is unavoidable to achieve the best performance in the classification of motor capabilities. Particularly, we found two best cases, the SVM_L applied to the PS_B dataset, and the SVM_G applied to the PC dataset, for whose perfect discrimination between the two groups was achieved, with sensitivity, specificity, accuracy, precision and F-measure equal to 1 in the FULL condition. In the end, there is not a significant difference in classifiers performance if using all the significant features of PS, which is the largest dataset, or if applying both the Bonferroni correction and correlation analysis to reduce the number of features as much as possible without losing relevant information (i.e., PC_B). The choice could be done according, for example, to the computational power and time available for the analysis. However, it is premature to define which is the best classifier and which is the best dataset to use and further extended studies should be carried out to allow generalization of the model. The threshold value chosen for the correlation analysis (i.e., rho = 0.85), for instance, even if quite high, has been already used in the literature [43,44,45] and, in this work, it has been selected also in a clinical perspective. Indeed, it allows maintaining much information from the original dataset, which is important in this work because the parameters are related to motor impairments characterizing PD. Hence, removing many features could lead to removing useful information for the motor evaluation as well. As in near future steps, the experimental dataset will be enlarged, it could be possible to have a definitive panoramic of the parameters that are worthy to be included or not and the threshold could be differently set, using also lower thresholds that can further reduce the dataset, favouring the simplification of the model and its generalization.

The focus of the present work, indeed, is the wearable system, composed of four inertial devices and processing algorithms, that we would propose for supporting the neurologist in the clinical assessment of motion for PD diagnosis and monitoring. The SensHand and SensFoot devices are prototypes that already reached TRL = 7, that means “system prototype demonstration in operational environment”, a preliminary validation of their accuracy respect to an optoelectronic gold-standard system was already performed [46] (e.g., for finger tapping task was obtained an average RMSE value equals to 2.12°, with an agreement between the two systems measured in terms of coefficient of determination equals to 0.987), and next steps will be carried out soon to obtain the certification of the system as a medical device.

Technically, the main challenges are to further improve the wearability of the system and automatize the processing algorithms for obtaining a system that could be easily used by specialized clinicians, general practitioners and, also, PD patients. The opportunity to use such a system at home, indeed, could positively impact on PD management, with benefits both for patients and healthcare systems, promoting home-monitoring through the empowerment of the patients and their caregivers. For the clinical application, we would overcome the assessment with the traditional MDS-UPDRS, which has low granularity, ranging from 0 to 4 for each task, so that the same score can be assigned to subjects with different problems [21]. Therefore, we propose to move towards a continuous scale that, merging the information derived from the extracted parameters by using machine learning techniques, could identify a status point for each patient and its evolution over the time. The main limitation of this work is that normative data are urgently needed to reach the achievement we aim to, thus we are working to enlarge the dataset of healthy controls and patients with different PD severity, to find reference values that clinically should validate our method. Another open challenge can be the use of SensHand and SensFoot to investigate the clinical assessment not only in PD patients but also in subjects with other forms of Parkinsonism, to enhance the assessment of differential diagnosis.

## 5. Conclusions

This work aimed at providing a wearable system, composed of inertial devices and processing algorithms, that could be a support for neurologists in the quantitative and objective assessment of motor performance for PD diagnosis and monitoring. Many kinematic parameters have been extracted during a motor evaluation protocol over 40 healthy subjects and 40 PD patients from upper and lower limbs, and the related algorithms were presented in detail. Statistical analysis and dimensionality reduction allowed to obtain four different datasets that were tested with five supervised learning methods to evaluate the accuracy of the system in distinguishing between healthy and patients. Three conditions were tested using parameters from lower, upper and all limbs. Excellent results were obtained with all the classifiers (average accuracy ranging from 0.936 for NB to 0.966 for SVM_G) and all the datasets (average accuracy ranging from 0.953 for PS, PC and PC_B to 0.960 for PS_B) over the three conditions, while best performances were achieved by linear SVM on PS_B dataset and Gaussian SVM on PC dataset (accuracy equal to 1.00) when analysing the FULL condition. The promising results confirm the need to use a wide set of exercises and parameters to have a complete motor assessment of the subjects. The idea is to develop an accurate and reliable system that could be easily applied in clinical practice to improve the diagnosis and management of Parkinson’s disease, to be used not only in hospital by professionals but also at home by empowered patients. Future works will focus on enlarging the dataset to reach normative data for clinical validation, including also subjects with Parkinsonism, and to improve the wearability of the system and the automation of the processing algorithms.

## Figures and Tables

**Table 1:** List of parameters measured for each task (lower limbs) both for the right and left sides.

	Exercise					
Parameter	TTHP	HTTP	HETO	HEHE	ROTA	GTAF
Number of movements	TT_Taps	HH_Taps	HT_Taps		RO_Strd	GT_Strd
Frequency	TT_Freq	HH_Freq	HT_FreqT HT_FreqH HT_FreqHT	HE_Freq	RO_Freq	GT_Freq
Max. Amplitude	TT_Exc	HH_Exc	HT_ExcT HT_ExcH			GT_Ang
Frequency Variability	TT_CVfreq	HH_CVfreq	HT_CVfreq			
Amplitude Variability	TT_CVexc	HH_CVexc	HT_CVexcT HT_CVexcH			
Time					RO_Time	GT_Time
Stride Time						GT_StrdT
Swing Time						GT_SWT
Stance Time					RO_STT	GT_STT
Relative Stance					RO_RS	GT_RS
Average Power				HE_Power		
Peak Power				HE_Peak		
IAV	TT_IAV	HH_IAV	HT_IAV	HE_IAV		

**Table 2:** List of parameters measured for each task (upper limbs) both for the right and left sides.

	Exercise					
Parameter	THFF	OPCL	PSUP	HRST ^1^	POST ^1^	GTAH
Number of movements	TF_Taps	OC_Taps	PS_Taps			GT_Taps
Frequency	TF_Freq	OC_Freq	PS_Freq	RT_FreqA RT_FreqG	PT_FreqA PT_FreqG	GT_HFreq
Max. Amplitude	TF_Exc	OC_Exc	PS_Exc			GT_Exc
Opening Velocity	TF_ωo	OC_ωo	PS_ωs			GT_ωf
Closing Velocity	TF_ωc	OC_ωc	PS_ωp			GT_ωb
Frequency Variability	TF_CVfreq	OC_CVfreq	PS_CVfreq			GT_CVfreq
Amplitude Variability	TF_CVexc	OC_CVexc	PS_CVexc			GT_CVexc
Average Power				RT_PwrA RT_PwrG	PT_PwrA PT_PwrG	
% Power [3.5–7.5] Hz				RT_Perc1A RT_Perc1G	PT_Perc1A PT_Perc1G	
% Power [8–12] Hz					PT_Perc2A PT_Perc2G	
IAV	TF_IAV	OC_IAV	PS_IAV	RT_IAV	PT_IAV	GT_IAV

^1^ In HRST and POST tasks, A is referred to parameters measured from acceleration data; G is referred to parameters measured from the gyroscope.

**Table 3:** Median and interquartile range (IQR) values for parameters extracted from lower limbs for both HC and PD. Significance of parameters in the four datasets (PS, PS_B, PC, PC_B) is marked.

	Left		Right		Significance			
Parameter	HC Median (IQR)	PD Median (IQR)	HC Median (IQR)	PD Median (IQR)	PS	PS_B	PC	PC_B
TT_Taps	33.0 (11.2)	29.0 (9.0)	38.5 (9.2)	31.0 (9.0)	X	X	X	X
TT_Freq	3.29 (1.17)	2.89 (0.92)	3.93 (0.95)	3.11 (0.98)	X	X		
TT_Exc	10.4 (5.3)	7.7 (5.8)	7.2 (5.4)	7.6 (5.4)				
TT_CVfreq	41.3 (25.4)	48.3 (23.6)	39.8 (18.1)	38.7 (22.8)				
TT_Cvexc	60.3 (35.1)	70.1 (27.4)	65.0 (23.5)	64.1 (27.4)				
TT_IAV	108.6 (4.2)	99.0 (17.7)	111.5 (4.5)	101.2 (20.7)	X	X	X	X
HH_Taps	38.0 (7.2)	31.7 (8.2)	40.0 (8.0)	32.2 (10.2)	X	X	X	X
HH_Freq	3.79 (0.74)	3.16 (0.94)	4.02 (0.79)	3.22 (1.06)	X	X		
HH_Exc	10.3 (6.6)	5.3 (8.8)	8.8 (6.3)	6.2 (5.8)	X		X	
HH_Cvfreq	53.0 (13.0)	56.8 (20.6)	54.8 (18.3)	54.5 (27.1)				
HH_Cvexc	80.6 (15.7)	85.7 (21.0))	84.4 (18.9)	82.6 (26.4)				
HH_IAV	110.6 (4.1)	106.3 (8.9)	112.2 (3.9)	106.6 (9.3)	X	X	X	X
HT_Taps	14.5 (3.5)	13.7 (3.2)	15.7 (4.0)	13.5 (3.5)	X	X	X	X
HT_FreqT	1.50 (0.37)	1.43 (0.32)	1.64 (0.46)	1.40 (0.36)	X	X		
HT_FreqH	1.51 (0.37)	1.44 (0.31)	1.64 (0.46)	1.41 (0.36)	X	X		
HT_FreqHT	3.17 (0.70)	2.97 (0.76)	3.35 (0.81)	2.76 (0.93)	X	X		
HT_ExcT	36.6 (7.7)	21.4 (13.7)	32.0 (10.9)	20.1 (9.7)	X	X	X	X
HT_ExcH	36.3 (8.3)	22.4 (12.6)	31.7 (11.4)	20.6 (9.3)	X	X		
HT_Cvfreq	40.8 (30.2)	61.5 (26.9)	41.3 (31.6)	53.1 (31.1)	X	X	X	X
HT_CvexcT	52.6 (48.2)	71.9 (38.4)	49.2 (44.8)	44.8 (39.9)	X	X		
HT_CvexcH	55.6 (45.8)	70.3 (41.5)	56.0 (50.0)	66.5 (32.9)	X			
HT_IAV	101.4 (4.9)	96.1 (14.7)	103.6 (3.9)	96.2 (14.0)	X	X	X	X
HE_Power	81.9 (32.4)	6.1 (16.6)	82.8 (37.5)	7.2 (12.5)	X	X	X	X
HE_Peak	111.1 (80.1))	127.8 (92.5)	13.2 (28.4)	13.3 (23.6)	X	X		
HE_Freq	3.86 (0.69)	3.59 (0.83)	4.30 (0.93)	3.83 (0.76)	X	X	X	X
HE_IAV	140.5 (26.4)	104.5 (11.8)	141.4 (25.8)	102.6 (11.2)	X	X		
GT_Time	11.2 (1.9)	13.4 (2.2)	11.9 (2.1)	13.7 (3.1)	X	X	X	X
GT_Strd	11.0 (1.8)	12.5 (2.3)	11.0 (1.8)	12.8 (3.0)	X	X	X	X
GT_Freq	0.95 (0.09)	0.94 (0.06)	0.96 (0.10)	0.94 (0.11)				
GT_StrdT	1.07 (0.11)	1.09 (0.07)	1.06 (0.11)	1.08 (0.13)				
GT_SWT	0.33 (0.03)	0.32 (0.03)	0.32 (0.03)	0.33 (0.03)				
GT_STT	0.74 (0.09)	0.76 (0.07)	0.75 (0.09)	0.77 (0.10)				
GT_RS	69.1 (1.7)	69.7 (1.9)	70.4 (2.4)	70.2 (2.1)	X		X	
GT_Ang	92.1 (9.8)	75.1 (14.0)	76.9 (11.0)	68.9 (13.4)	X	X	X	X
RO_Time	2.5 (0.6)	2.3 (1.0)	3.7 (1.7)	3.7 (1.7)	X	X	X	X
RO_Strd	3.0 (1.0)	3.0 (0.5)	4.5 (1.2)	4.5 (1.5)	X	X	X	X
RO_Freq	1.39 (0.29)	1.34 (0.40)	1.20 (0.33)	1.25 (0.35)	X		X	
RO_STT	1.07 (0.48)	1.01 (0.67)	1.65 (1.23)	1.76 (1.30)	X	X		
RO_RS	44.1 (11.3)	44.4 (13.6)	48.6 (14.5)	48.7 (11.9)	X			

**Table 4:** Median and Interquartile Range (IQR) values for parameters extracted from upper limbs for both HC and PD. Significance of parameters in the four datasets (PS, PS_B, PC, PC_B) is marked.

	Left		Right		Significance			
Parameter	HC Median (IQR)	PD Median (IQR)	HC Median (IQR)	PD Median (IQR)	PS	PS_B	PC	PC_B
PS_Taps	23.0 (7.5)	16.0 (10.7)	23.8 (9.8)	15.5 (9.0)	X	X	X	X
PS_Freq	2.36 (0.75)	1.62 (1.07)	2.37 (1.09)	1.50 (0.95)	X	X		
PS_Exc	157.1 (39.7)	107.1 (44.5)	149.6 (36.8)	122.8 (41.9)	X	X	X	X
PS_ωp	641.5 (139.9)	349.5 (193.2)	647.4 (163.5)	334.2 (218.7)	X	X		
PS_ωs	715.2 (233.2)	338.5 (206.8)	695.1 (215.5)	324.9 (196.9)	X	X	X	X
PS_CVfreq	24.5 (16.3)	24.3 (22.1)	23.2 (12.9)	21.7 (17.9)				
PS_CVexc	24.2 (31.4)	27.1 (37.0)	22.1 (15.8)	29.6 (26.7)				
PS_IAV	155.8 (55.5)	109.7 (11.0)	150.6 (53.4)	107.5 (16.5)	X	X		
OC_Taps	34.0 (6.8)	21.5 (15.5)	36.0 (9.8)	21.5 (13.3)	X	X	X	X
OC_Freq	3.39 (0.68)	2.10 (1.50)	3.58 (1.00)	2.14 (1.32)	X	X		
OC_Exc	104.3 (44.1)	116.4 (77.5)	89.7 (50.7)	103.3 (73.3)				
OC_ωo	597.3 (211.6)	458.5 (326.9)	554.5 (215.6)	433.3 (268.0)	X			
OC_ωc	706.5 (249.9)	435.5 (420.0)	637.8 (257.1)	464.8 (361.9)	X	X	X	X
OC_CVfreq	23.4 (12.3)	30.1 (28.4)	25.4 (14.0)	26.1 (24.9)	X		X	
OC_CVexc	44.8 (26.4)	50.6 (34.2)	56.6 (23.3)	41.7 (35.9)				
OC_IAV	258.5 (81.8)	144.7 (97.8)	241.7 (66.7)	136.2 (90.3)	X	X	X	X
TF_Taps	44.3 (11.5)	29.3 (16.5)	46.5 (12.0)	31.5 (17.5)	X	X	X	X
TF_Freq	4.45 (1.20)	2.94 (1.71)	4.69 (1.21)	3.17 (1.76)	X	X		
TF_Exc	23.6 (21.5)	24.5 (25.9)	15.9 (17.9)	20.9 (26.6)				
TF_ωo	169.6 (119.6)	110.9 (104.3)	117.0 (104.1)	109.1 (114.6)				
TF_ωc	201.8 (138.2)	119.5 (135.8)	144.4 (123.2)	127.3 (132.7)	X		X	
TF_CVfreq	26.3 (19.1)	41.3 (37.9)	24.8 (22.5)	49.1 (35.3)	X	X	X	X
TF_CVexc	73.9 (23.7)	75.0 (42.9)	82.1 (21.7)	83.7 (32.4)				
TF_IAV	147.7 (31.7)	114.0 (39.7)	129.3 (27.6)	112.3 (26.6)	X	X	X	X
GT_Taps	12.8 (1.5)	13.0 (2.5)	12.5 (1.8)	13.8 (3.5)	X	X	X	X
GT_HFreq	0.96 (0.12)	0.97 (0.13)	0.96 (0.10)	0.97 (0.18)				
GT_Exc	73.7 (38.1)	41.4 (42.0)	74.3 (35.7)	38.8 (29.6)	X	X		
GT_ωf	77.8 (40.6)	45.8 (44.2)	64.3 (39.6)	31.0 (30.7)	X	X		
GT_ωb	55.4 (24.9)	49.6 (27.0)	43.4 (31.9)	58.9 (27.4)	X		X	
GT_CVfreq	13.3 (8.8)	21.7 (50.4)	11.8 (16.7)	29.3 (46.0)	X		X	
GT_CVexc	41.3 (21.7)	24.1 (26.3)	38.1 (19.7)	18.3 (17.3)	X	X	X	X
GT_IAV	135.7 (25.3)	142.4 (16.9)	127.0 (15.1)	143.5 (29.3)	X	X	X	X
RT_PwrA	0.0013 (0.0005)	0.0018 (0.0009)	0.0017 (0.0005)	0.0019 (0.0009)	X		X	
RT_FreqA	6.42 (3.78)	6.57 (3.98)	6.84 (4.17)	5.79 (3.22)				
RT_Perc1A	29.3 (4.8)	31.0 (4.0)	29.4 (6.5)	31.3 (7.4)	X		X	
RT_IAV	97.7 (2.2)	103.3 (8.7)	98.7 (3.8)	103.6 (5.3)	X		X	
RT_PwrG	0.052 (0.604)	0.675 (0.459)	0.048 (0.572)	0.749 (1.084)	X	X	X	X
RT_FreqG	5.42 (2.61)	4.93 (2.12)	5.23 (3.13)	5.28 (2.62)				
RT_Perc1G	32.6 (8.2)	38.2 (14.1)	32.5 (7.0)	37.1 (18.1)	X		X	
PT_PwrA	0.017 (0.015)	0.022 (0.031)	0.015 (0.012)	0.019 (0.026)				
PT_FreqA	7.59 (4.03)	7.62 (2.40)	8.30 (1.88)	7.40 (2.25)	X		X	
PT_Perc1A	17.3 (8.5)	30.7 (16.0)	22.7 (10.8)	28.9 (19.0)	X	X	X	X
PT_Perc2A	36.4 (11.8)	30.6 (11.5)	35.0 (11.9)	28.6 (17.7)	X		X	
PT_PwrG	1.39 (0.29)	1.34 (0.40)	1.20 (0.33)	1.25 (0.35)	X		X	
PT_FreqG	5.59 (5.18)	5.84 (3.23)	7.15 (3.76)	5.76 (3.44)				
PT_IAV	100.1 (2.6)	103.4 (7.2)	99.6 (3.4)	101.6 (9.1)	X	X	X	X
PT_Perc1G	25.0 (11.7)	33.7 (20.5)	22.3 (7.3)	31.7 (20.5)	X	X	X	X
PT_Perc2G	27.0 (10.5)	22.7 (15.1)	30.5 (15.2)	23.8 (20.8)	X		X	

**Table 5:** Classification Results for Post-Significance and Post-Correlation datasets.

	PS					PC				
FEET	RF	SVM_L	SVM_G	SVM_P	NB	RF	SVM_L	SVM_G	SVM_P	NB
Recall	0.900	0.900	0.850	0.900	0.850	0.925	0.925	0.950	0.900	0.850
Specificity	0.950	0.900	1.000	0.900	1.000	0.950	0.900	0.925	0.975	1.000
Accuracy	0.925	0.900	0.925	0.900	0.925	0.938	0.913	0.938	0.938	0.925
Precision	0.947	0.900	1.000	0.900	1.000	0.949	0.902	0.927	0.973	1.000
F_measure	0.923	0.900	0.919	0.900	0.930	0.937	0.914	0.938	0.939	0.930
**HANDS**										
Recall	0.975	0.975	0.975	0.975	0.975	0.975	0.975	1.000	0.950	0.950
Specificity	0.975	1.000	1.000	0.975	0.900	1.000	0.975	0.975	0.925	0.900
Accuracy	0.975	0.988	0.988	0.975	0.938	0.988	0.975	0.988	0.938	0.925
Precision	0.975	0.988	1.000	0.975	0.907	1.000	0.975	0.976	0.927	0.905
F_measure	0.975	0.987	0.987	0.975	0.939	0.987	0.975	0.988	0.938	0.926
**FULL**										
Recall	0.975	0.975	0.975	0.950	0.900	0.950	0.925	1.000	0.950	0.925
Specificity	1.000	1.000	1.000	1.000	0.950	1.000	1.000	1.000	1.000	0.925
Accuracy	0.975	0.988	0.988	0.975	0.925	0.975	0.963	1.000	0.975	0.925
Precision	1.000	1.000	1.000	1.000	0.947	1.000	1.000	1.000	1.000	0.925
F_measure	0.976	0.987	0.987	0.976	0.926	0.976	0.961	1.000	0.976	0.925

**Table 6:** Classification Results for Post-Significance and Post-Correlation datasets after Bonferroni correction.

	PS_B					PC_B				
FEET	RF	SVM_L	SVM_G	SVM_P	NB	RF	SVM_L	SVM_G	SVM_P	NB
Recall	0.925	0.900	0.900	0.975	0.850	0.900	0.950	0.900	0.950	0.850
Specificity	0.950	1.000	1.000	0.925	1.000	0.950	0.950	0.950	0.975	1.000
Accuracy	0.938	0.950	0.950	0.950	0.925	0.925	0.950	0.925	0.963	0.925
Precision	0.949	1.000	1.000	0.929	1.000	0.947	0.950	0.947	0.974	1.000
F_measure	0.937	0.947	0.947	0.951	0.930	0.923	0.950	0.923	0.963	0.930
**HANDS**										
Recall	0.850	0.950	0.950	0.925	0.950	0.925	0.900	0.925	0.900	0.975
Specificity	0.975	0.975	0.975	1.000	0.975	1.000	0.950	0.975	1.000	0.975
Accuracy	0.913	0.963	0.963	0.963	0.963	0.963	0.925	0.950	0.950	0.975
Precision	0.971	0.963	0.974	1.000	0.974	1.000	0.926	0.974	1.000	0.975
F_measure	0.907	0.962	0.962	0.964	0.963	0.961	0.923	0.949	0.952	0.975
**FULL**										
Recall	0.950	1.000	0.975	0.975	0.950	0.950	0.975	0.975	0.950	0.925
Specificity	0.975	1.000	1.000	1.000	0.975	1.000	1.000	1.000	1.000	0.925
Accuracy	0.988	1.000	0.988	0.988	0.963	0.975	0.988	0.988	0.975	0.925
Precision	0.974	1.000	1.000	0.974	0.974	1.000	1.000	1.000	1.000	0.925
F_measure	0.988	1.000	0.987	0.988	0.963	0.976	0.987	0.987	0.976	0.925

## Supplementary Materials

The following are available online at https://www.mdpi.com/1424-8220/20/9/2630/s1.

## Appendix A

The list of the main abbreviations used in the manuscript are reported in Table A1.

**Table A1:** List of Abbreviations.

Abbreviation	Description
PD	Parkinson’s Disease
HC	Healthy subjects of control
QoL	Quality of Life
MDS	Movement Disorder Society
UPDRS	Unified Parkinson’s Disease Rating Scale
HY	Hoehn & Yahr
TRL	Technological Readiness Level
IMU	Inertial Measurement Unit
ZUPT	Zero Velocity Update
THFF	Thumb Forefinger Tapping
OPCL	Hand Opening/Closing
PSUP	Hand Pronation/Supination
HRST	Hand Resting Tremor
POST	Hand Postural Tremor
TTHP	Toe Tapping Heel Pin
HEHE	Leg Agility
HTTP	Heel Tapping Toe Pin
HETO	Heel-Toe Tapping
ROTA	Rotation
GTAF	Gait
GTAH	Arms swing while walking
PS dataset	Dataset Post Significance
PS_B dataset	Dataset Post Significance with Bonferroni Correction
PC dataset	Dataset PS and Post Correlation
PC_B dataset	Dataset PS and Post Correlation with Bonferroni Correction
SVM	Support Vector Machine
SVM_P	Support Vector Machine with Polynomial kernel
SVM_L	Support Vector Machine with Linear kernel
SVM_G	Support Vector Machine with Gaussian kernel
RF	Random Forest
NB	Naïve Bayes
FEET	Condition including parameters from lower limbs only
HANDS	Condition including parameters from upper limbs only
FULL	Condition including all the measured parameters
